# Macrophage Activation Syndrome and COVID 19: Impact of MAPK Driven Immune-Epigenetic Programming by SARS-Cov-2

**DOI:** 10.3389/fimmu.2021.763313

**Published:** 2021-10-01

**Authors:** Roshan Kumar Roy, Uttam Sharma, Mishi Kaushal Wasson, Aklank Jain, Md. Imtaiyaz Hassan, Hridayesh Prakash

**Affiliations:** ^1^ Amity Institute of Virology and Immunology, Amity University, NOIDA, Udham Singh Nagar, India; ^2^ Department of Zoology, Central University of Punjab, Bathinda, India; ^3^ Centre for Interdisciplinary Research in Basic Sciences, Jamia Millia Islamia, Jamia Nagar, New Delhi, India

**Keywords:** COVID-19, macrophage, TLRs (toll-like receptors), inflammasome, miRNA, lncRNAs, MAPK, immune polarization

## Introduction

The current coronavirus disease 2019 (COVID-19), which is caused by severe acute respiratory syndrome coronavirus 2 (SARS-CoV-2), has the worst affected the entire population on the earth (1, 2). This is currently a major concern for the global health care system, as declared by the World Health Organization (WHO). Ample pieces of evidences suggested the idiopathic association of the SARS-CoV-2 with many diseases in COVID-19 cases. Given the aberrant immunopathology of COVID-19, a single approach may not be sufficient to control the disease effectively. Severely infected patients displaying acute respiratory distress syndrome (ARDS) need additional modalities for their management (3). This could be due to the host’s epigenetic programming of infected macrophages, which may be responsible for negative prognosis and inadequate response to the current therapeutic regimen for controlling disease manifestation.

SARS-CoV-2 enters the host cells *via* ACE-II receptor and triggers the secretion of the copious amount of IL-6;promote pulmonary fibrosis and Th2/17 programming of lungs, leading to severe lung infection in COVID-19 patients. SARS-CoV-2 interacts and tweaks all kind of cells like epithelium, macrophages, dendritic cells, and T cells and exploit them in a way that supports its replication for progression of the disease.

Out of these, uncontrolled activation of macrophages (also known as double edge component of immunity) leads to Macrophage activation syndrome which is responsible for acute respiratory distress syndrome (ARDS) and subsequent death of COVID-19 patients (4, 5). This is mainly characterized by the increased infiltration of committed ${\text{F}}_{C}^{\text{N}}1^{+}$ macrophages and their Th2/Th17 programming leading to mortality. Once derailed, hyperactive macrophages secrete high levels of IFN‐γ, IP-10 (IP‐10), IL-6, IL-17, TNF-α along with TGF-β and IL-10/23, leading to the Th2/Th17 programming in the infected lungsof severe cases of COVID-19 (6).

**Figure 1:**
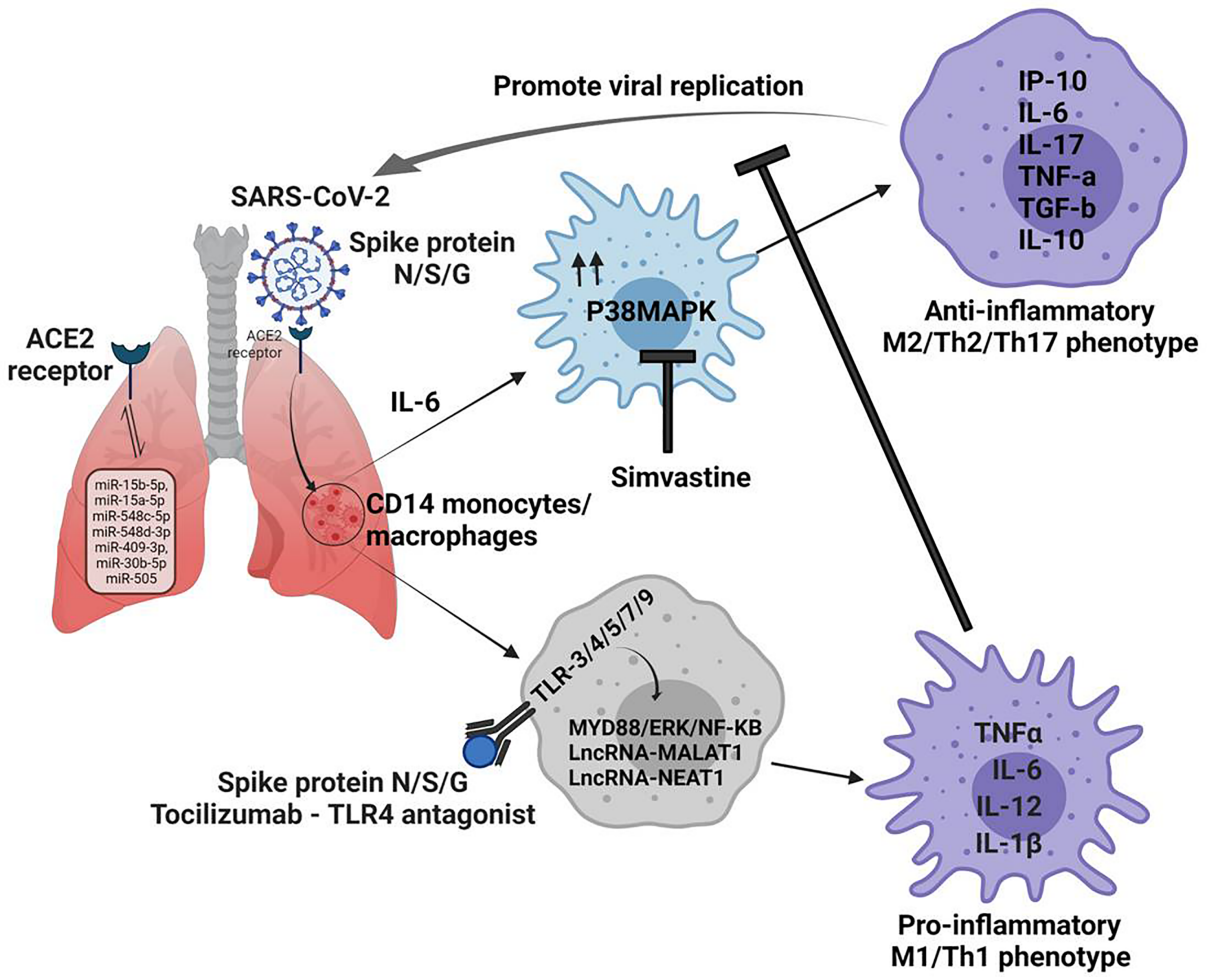
Non-coding RNAs regulates macrophage plasticity during the pathogenesis of Covid 19 disease. 1. N/S/G spike proteins bind to ACE2 receptors on lung cells and determine the entry of the SARS-CoV-2 virus. 2. miRNA can be direct targets since they can regulate the expression of ACE2 in various organs. 3. Infection produces an copius amount of IL-6, which drives the fate of CD14 monocytes/macrophages towards M2 phenotype *via* MAPK signaling, which promotes viral replication. 4. In view of this virus eliminating inflammatory niche could be achievd by promting M1 phenotype in TLR depenedent MYD88/ERK/NF-Kb pathway. 5. This could be fosterd by application of MAPK inhibitors like simvastatin in conjuction of TLR antagonist which can help immune cells to curb SARS-CoV-2 in the host effectively.

At molecular levels, this is accompanied by the activation of inflammasome pathways which are important forTh17 programming of tissue. Activated CD14+ monocytes phagocytose dead neutrophils and promotes NETosis in the lung. This promotes Th2 bias, decreases lymphocyte/neutrophils ratio and increases the risk of COVID-19 patients for death. Given this, *in situ* reprogramming Th2/Th17 programmed macrophages towards their M1 phenotype is expected to afford protective immunity in COVID-19 cases (4) as shown in **Figure 1**.

Committed macrophages rely upon Toll-like Receptors (TLRs) and associated pathways, the guardian for Th1/2/17 effector responsesduring any infection, including SARS-CoV-2 (7). Among various TLRs on macrophages, TLR-4, 5, 3, 7,and 9 actively sense spike proteins (N, S or G) or mRNA of NSP-10, S2, and E proteins of SARS-CoV-2 and promote M1 polarization of macrophages (8). Apart from ACE-2, the spike protein of SARS-CoV-2 uses TLR-2, 4 and 5 signaling pathways also *via* MyD88 and triggers Th1 effector response through NF-κB and ERK signaling cascade (9). Given this, tweaking TLR signaling like TLR5 can restore or promote Th1 response in derailed macrophages in COVID-19 patients. Indeed, a recent report suggests that conjunction therapy with antivirals and TLR-7 agonists may benefit patients (7) who are believed to harbor Th2/17 programmed macrophage. Similarly, the application of Tocilizumab and TLR-4 antagonists is expected to promote M1 re-polarization of derailed macrophage in patients with severe disease displaying ARDS.

Several intracellular pathways like nk-kb/STAT/and p38MAPK are essential for the immune polarization of macrophages during infection and cancer. p38MAPK pathway is one of the host factors implicated in lung and heart injury in COVID-19 patients (10, 11). P38MPAK landscape is decisive for sterile inflammatory responses, desmoplastic reactions, T cell exhaustion, and epigenetic programming of severely infected COVID-19 cases. P38 MAPK controls macrophage plasticity *via* promoting ER stress, unfolded protein responses, and glucose intolerance which are associated with energy imbalance in the infected host. Since SARS-CoV-2 directly up-regulates p38 activity for promoting its replication in epithelium and macrophages (12), we presume that hyper-activation of p38MAPK may contribute to Th2 bias in these macrophages and aberrant inflammation in the lung.

SARS-CoV-2 regulates P38MAPK signaling in multiple ways to support its replication, one of the prominent mechanisms is downregulation of *ACE2* activity, which negatively regulate expression of *ICAM-1* (intercellular adhesion molecule-1), *VCAM-1* (vascular cell adhesion molecule-1) (13) and *NF-KB* activation (14) leading to Th2 bias in the host. Loss of *ACE2* function leads to enhanced concentration of intracellular Angiotensin 2, which directly activates P38MAPK (10) in the host, leading to Th17 response in the host (15). This progress to ARDS (acute respiratory distress syndrome) and myocarditis are primary reasons for death in critically infected patients (16). Several studies with severely infected patients suggested that SARS-CoV-2 promotes degradation of DUSPs (dual-specificity phosphatase) transcripts, this promotes *P38MAPK* hyperactivation (17) in the host. Besides ACE2 and DUSPs, SARS-CoV-2 also triggers TAB1 (TGF-β activated kinase 1 (MAP3K7)-bindingprotein 1) mediated P38A auto-phosphorylation and P38MAPK hyper-activation, adding to the reason for increased MAPK activity in the infected cells. Studies with several MAPK inhibitors like SB203580 (18), Losmapimod (19) and Dilmapimod (20) have shown promising results in mitigating pathogenic inflammation in COPD patients and advocated their potential application in hashing SARS-CoV-2 burden. Therefore targeting p38MAPK could be of direct interest in controlling viral burden and M1 retuning of infected macrophages *viz-a-viz* mitigating T- cell exhaustion in patients.

Apart from activating several cytoplasmic signaling pathways, p38MAPK also activate the expression of various transcription factors. Recent studies have provided compelling evidence that activated MAPK influence the expression of differentially expressed mi/lncRNAs,which are important for sterile inflammation and M2/Th2 polarization of macrophages. Most intriguingly, the lncRNA landscape is proposed as a prognostic factor responsible for the severity of COVID-19 cases (21). Among pool of miRNAs; miR-15b-5p, miR-15a-5p, miR-548c-5p, miR-548d-3p, miR-409-3p, miR-30b-5p and miR-505 have been validated as potent targets for controlling SARS-CoV-2 infection (22). These miRNAs regulate the expression of ACE-2 in various organs, including the kidney, heart, blood vessels, and lungs which are important for COVID-19 pathophysiology (23). Other than this, several LncRNA like *WAKMAR2*, *EGOT*, *EPB41L4A-AS1*, *ENSG00000271646*, *MALAT1* and *NEAT1 *are known to contribute to skewing the immune response against SARS-CoV-2 infection (24, 25).

Overexpression of *NEAT1* stabilizes the mature caspase-1 to promote interleukin-1β production and modulate inflammasome activation (26), which is associated with Th2/17 programming of immune cells like macrophages. *MALAT1* promotes Th1 effector responses and apoptosis in airway epithelial cells conditioned DCs and cardiac cells (6, 27) *via* miR-125b and p38MAPK/NF-κB pathways (7). This loop is potentially involved in the maturation and pro-inflammatory programming of CD14+/Gr-1-/iNOs+ M1 macrophages, which is essential for the adaptive immunity of the host.

## Major Perspective

Lowering p38MAPK with specific inhibitors like simvastatinin conjunction with TLR antagonist and Tocilizumab is anticipated to be a prudent approach for augmenting immunity of COVID-19 infected cases. The uncontrolled systemic inflammatory response and cytokine storm is the main mechanism of ARDS caused by the excessive release of interferon, interleukins, TNF-α and chemokines. Thus, it was proposed that statins (28), which are well known for their anti-inflammatory effects,could treat MERS-CoV infection and perhaps COVID-19 patients (29) as well. However, statins in COVID-19 patients sometimes increase the risk and severity of myopathies and acute kidney injury (29). On the other hand, statin therapy increases liver enzymes, leading to severe complications in the COVID-19 patients (30). Thus, guideline-directed statin therapy in COVID-19 patients is necessary.

It was proposed that early intervention with interleukin-6 receptor blockade by Tocilizumab could effectively control the progression to hypoxemic respiratory failure or death of severe COVID-19 patients (31). There are conflicting results obtained for tocilizumab in COVID-19 patients. Several treatment lines suggest that using a monoclonal antibody against IL-6 is an attractive strategy to manage severe COVID-19 as Tocilizumab has the potential to reduce mortality and the need for mechanical ventilation (32, 33). However, a clinical trial on 243 patients revealed that tocilizumab was not effective for preventing death in moderately ill hospitalized COVID-19 patients (34). In a recent study on the hospitalized COVID-19 patients, although tocilizumab reduced the progression to the composite outcome of mechanical ventilation, however could not improve their survival (35). Besides, this targeting miRNA which modulates the expression of ACE2 receptor activities, can also be of significant value to currently explored therapeutics/interventions. This conjuction approach is expected to enhance the sensitivity of infected host cells for currently employed drugs. Taken together, above interventions would help in curbing the SARS-CoV-2 virus for the effective management of COVID-19 disease.

## Author Contributions

Conceptualization and conceiving of idea, HP. Writing, AJ, RR, MW, and HP. Resources, AJ, MH, HP, and US. Editing of manuscript, HP. All authors contributed to the article and approved the submitted version.

## Funding

Funding for this study was provided by the Department of Health and Research, Government of India (GIA/2020/000414) to HP.

## Conflict of Interest

The authors declare that the research was conducted in the absence of any commercial or financial relationships that could be construed as a potential conflict of interest.

## Publisher’s Note

All claims expressed in this article are solely those of the authors and do not necessarily represent those of their affiliated organizations, or those of the publisher, the editors and the reviewers. Any product that may be evaluated in this article, or claim that may be made by its manufacturer, is not guaranteed or endorsed by the publisher.
